# Anatomical observation, classification, fracture and finite element analysis of the posterior process of the Asian adult talus

**DOI:** 10.1186/s13018-022-03345-5

**Published:** 2022-10-08

**Authors:** Han Yang, Liqing Liao, Fan Xue, Yikai Li, Guanyu Hu

**Affiliations:** 1 School of Traditional Chinese Medicine, Southern Medical University, No. 1838, North of Guangzhou Great Road, BaiYun District, Guangzhou, 510515 Guangdong Province China; 2 The Third Affiliated Hospital of Southern Medical University, No. 183, West of Zhongshan Avenue, Tianhe District, Guangzhou, 510630 Guangdong Province China

**Keywords:** Anatomy, Talus, Posterior process of the talus, Talus fracture, Finite element model

## Abstract

**Background:**

Fractures of the posterior process of the talus are rarely seen and frequently overlooked. In our study, anatomical observation and classification of the posterior process of the talus were carried out, and related imaging and finite element methods were combined. The study aimed to observe and provide anatomical data related to posterior process of talus in Asian adults and explore the potential relationships between the different types with fracture of posterior process of talus.

**Methods:**

Combined with the anatomical morphology and imaging data, the posterior process of talus was divided into four types, and the incidence and fracture situation were statistically analyzed. The finite element models of four different types of talus processes were established and verified, and the stress and strain were simulated and analyzed.

**Results:**

The total incidence of the posterior process of the talus was 97.47%. The proportions of the four types were neck-like 10.13%, flat 36.29%, pointy 12.66% and round blunt 38.39%. The overall incidence of bone cracks of the posterior process of the talus was 4.98%; the most common type was neck-like type. Compared with the value on the other types, the maximum von Mises stress increased by 67.66%, 83.90% and 111.18% on the neck-like posterior process of talus respectively.

**Conclusions:**

It is speculated that different types of the posterior process of the talus may be related to the probability of fracture, and it may be better to consider different treatment strategies for different types of fractures.

## Introduction

The talus is a critical link between the leg and the foot, playing an important role in normal ambulation [1]. The posterior process of the talus (PPOT) is a bony process at the posterior part of the talus body, consisting of the lateral and medial tubercles, which are separated by a groove for the flexor hallucis longus tendon situated in between the two tubercles [2, 3]. The lateral tubercle is the larger of the two projects and attached to the posterior talofibular ligament and the posterior talofibular ligament [4]. The medial tubercle is smaller and is the insertion point which provides attachment to the posterior third of the deltoid ligament superiorly and the medial limb of the bifurcate talocalcaneal ligament inferiorly [5].

Talus fractures constitute 0.32% of all fractures and usually involve the talar head and neck, PPOT fractures are relatively rare clinically, accounting for 0.90–0.14% of total body fractures and 3–6% of foot fractures [3, 6].The PPOT fracture was most likely first described and reported by Shepherd in 1882, and the case series of the PPOT fractures was published by Cedell in 1974 [7, 8]. Fracture of the PPOT includes two tubercles, divided into total PPOT fracture, lateral tubercle fracture (Shepherd fracture) and medial tubercle fracture (Cedell fracture); according to fracture site, the fractures more commonly involve the lateral tubercle [4, 6, 8]. Fracture of the PPOT is mainly caused by direct and indirect trauma factors, leading to a ligamentous avulsion or direct loading. The fracture may lead to detachment of the posterior talofibular and part of the deltoid ligament and cause damage in two joints (the posterior facet of the subtalar joint and the tibiotalar joint) increasing the risk of osteoarthritis and persistent pain, causing potentially ankle instability and reduced function [3, 4].

Fractures of the PPOT are rare and may be easily missed, CT scans are helpful of this fracture rather than plain radiographs [9]. Delayed diagnosis and treatment can potentially lead to adverse outcomes, resulting in long-term pain, disability, nonunion and degenerative changes [5, 9]. It is essential to understand these anatomical features of the talus; the study aimed to observe and provide anatomical data related to PPOT in Asian adults by investigating the incidence, length and type of the PPOT; to explore the potential relationships between the different types with fracture of PPOT; and to provide a reference for clinical, scientific research and teaching of modern medicine.

## Materials and methods

**Fig. 1:**
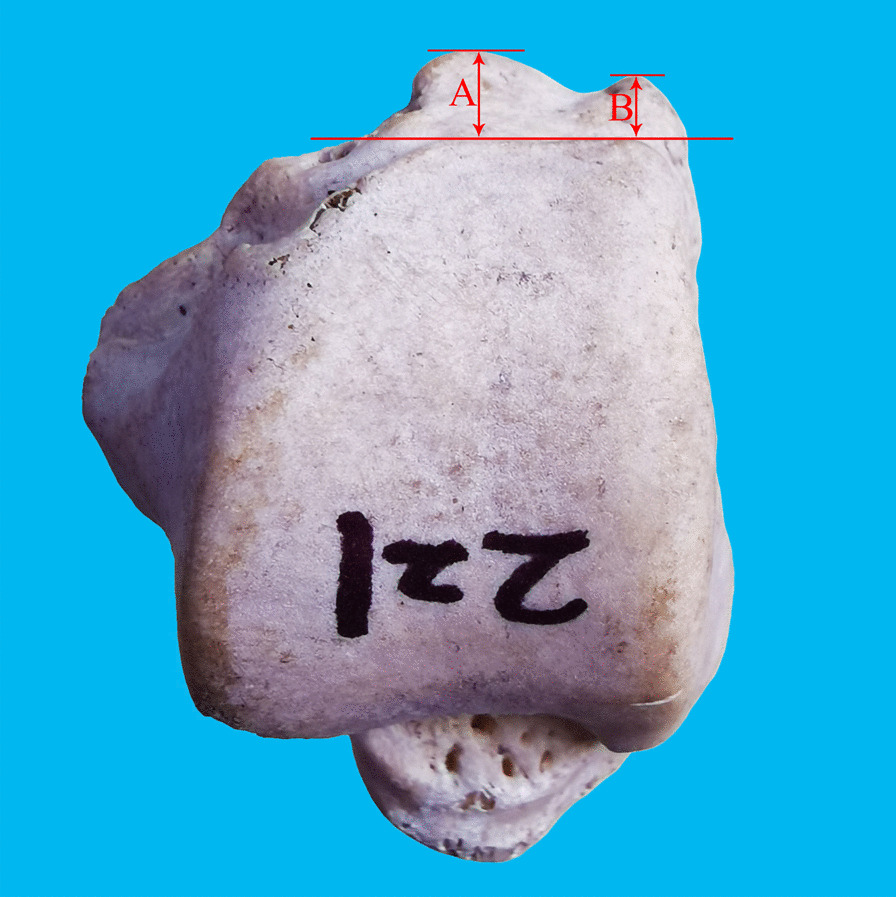
Measurement of the length of the medial and lateral tubercles of the posterior process of the talus. **A** Length of the lateral tubercle: the distance from the farthest point of the lateral tubercle to the plane of the vertical ground through the lowest point of the talar trochlea. **B** Length of the medial tubercle: the distance from the farthest point of the medial tubercle to the plane of the vertical ground through the lowest point of the talar trochlea

A total of 474 dry Asian adult talus specimens were observed and measured after excluding talus damage, lesions and malformations. The sex of the specimens was unknown; either no age identification was made. The incidence and shape of the posterior process of the talus were observed by visual inspection, and the length of the medial and lateral tubercles of the posterior process of the talus was measured (Fig. 1) using a digital Vernier caliper with an accuracy of 0.01 mm (Wuxi Kaibaoding Tools Co., Ltd., China). All the above items were measured three times independently by four researchers. After the measurement, the digital image acquisition (D610 camera, Nikon) and image processing (Photoshop 2020, Illustrator 2020, Adobe) were performed. All methods in the current study complied with the Declaration of Helsinki.

### Model construction

On the basis of a retrospective study of the hospital imaging system, we collected a number of computed tomography (CT) images of the ankle joint and selected four typical CT images with the four different types of the posterior process of talus mentioned above. Four ankle joint finite element (FE) models with different types of the lateral tubercle of posterior process of talus were constructed (neck-like, pointy, round blunt and flat respectively).

**Fig. 2:**
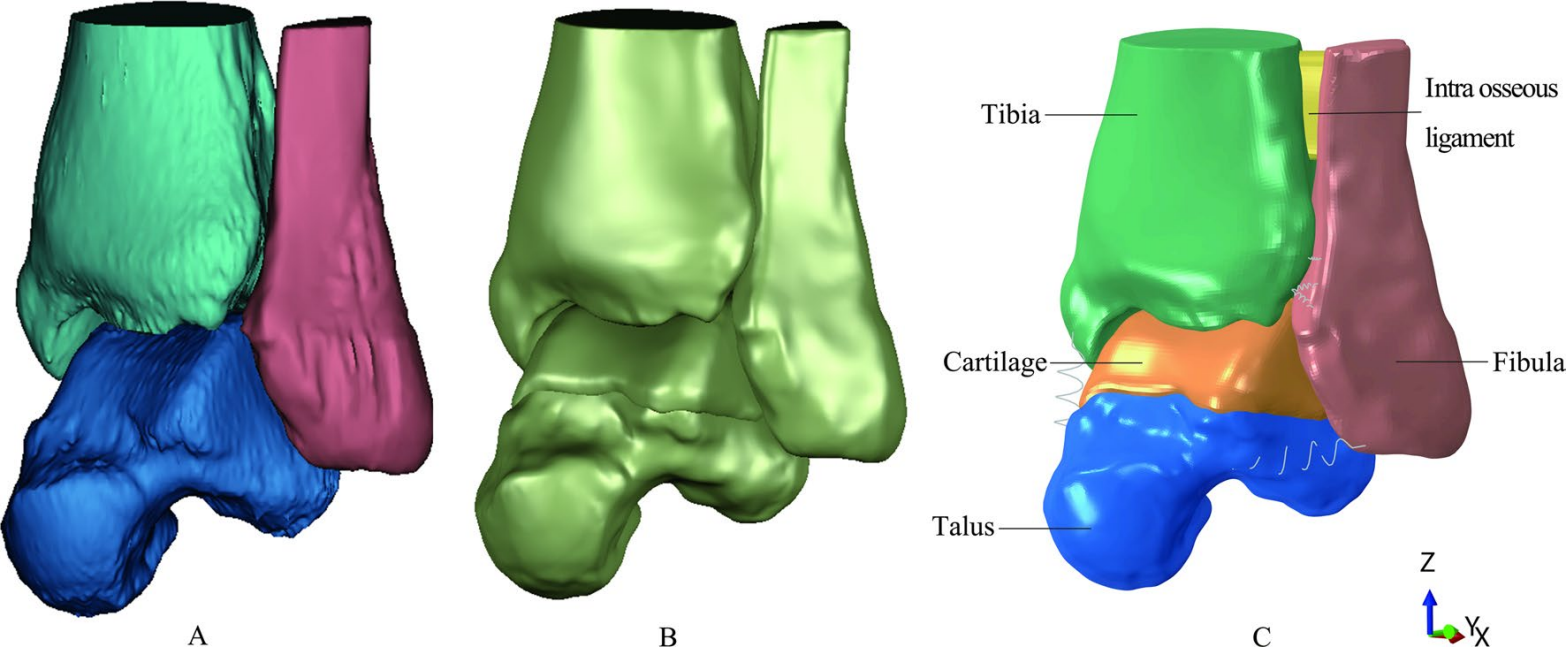
The ankle joint models we constructed. **A** The basic geometry of talus, tibia and fibula by Mimics software; **B** The high-quality nonuniform rational B-splines (NURBS) surface model by Geomagic Wrap software; **C** The intact finite element model by Solidworks and ABAQUS softwares

Specifically, the CT scans were performed with multidetector scanner (Philips, Brilliance 64, The United States). CT parameters were as follows: tube voltage of 100 kVp and YC filter; tube current of 150–200 mAS with automatic exposure control; slice thickness of 1 mm; spacing between slices 0.5 mm. The scanned CT data, saved in DICOM format, were imported into Mimics19.0 software (Materialise, Leuven, Belgium) to construct the basic three-dimensional (3D) surface geometry of talus, tibia and fibula (Fig. 2A). Then, the Geomagic Wrap 2017 software (Raindrop, Marble Hill, New York) was used to obtain the cartilage on the surface of talus (thickness of 1.4 mm) and finally output the high-quality nonuniform rational B-splines (NURBS) surface model (Fig. 2B). After that, intra-osseous ligament between the tibia and fibula was constructed based on its anatomical position using Solidworks 2017 software (Dassault Systems SA, Waltham, Massachusetts). The specific anatomical location of other ligaments is shown in Table 1 and the model was confirmed with colleagues with expertise in anatomy. Finally, all the entities were imported to ABAQUS 2020 (Simulia/Dassault Systèmes, Vélizy-Villacoublay, France) in SAT format.

**Table 1:** Material properties and element types

Component	Element type	Material type	Material parameters	Anatomical location
Cortical bone	Quadratic tetrahedral solid (C3D10M)	Elastic	*E* = 17,000 MPa *υ* = 0.3	–
Cancellous bone	Quadratic tetrahedral solid (C3D10M)	Elastic	*E* = 400 MPa *υ* = 0.3	–
Cartilage	Quadratic tetrahedral solid (C3D10M)	Elastic	*E* = 10 MPa *υ* = 0.45	–
Intra osseous ligament	Linear hexahedral solid (C3D8R)	Elastic	*E* = 20 MPa *υ* = 0.45	Between the distal tibia and fibula
Anterior talofibular ligament	Spring	Elastic	Stiffness = 14.2 N/mm	Fibular obscure tubercle—Talar obscure tubercle
Posterior talofibular ligament	Spring	Elastic	Stiffness = 16 N/mm	Posterior margin of the lateral malleolus—Lateral process of the talus
Anterior tibiotalar Ligament	Spring	Elastic	Stiffness = 115 N/mm	Anterior margin of the medial malleolus—Anterior border of medial malleolus articular surface of talus
Posterior tibiotalar Ligament	Spring	Elastic	Stiffness = 115 N/mm	Distal center of the intercollicular groove of the medial malleolus—Posterosuperior margin of the medial talar body
Anterior tibiofibular ligament	Spring	Elastic	Stiffness = 78 N/mm	Distal tibia—Anterior margin of the distal fibula
Posterior tibiofibular ligament	Spring	Elastic	Stiffness = 101 N/mm	Lateral malleolus—Posterolateral tibia tubercle

In ABAQUS 2020, the related ligaments around the ankle joint were constructed using the spring unit. Material properties of different parts were obtained from studies in the literature [10–12] (Table1). In this study, all the parts in the model were regarded as homogeneous, continuous and isotropic. We set binding relationships between the cartilage and the talus, as well as the cortical bone and cancellous bone. Frictionless surface-to-surface contact was used in our model. We used quadratic tetrahedral element to mesh the different parts. Also, we performed a convergence check to ensure that the mesh density was acceptable. The convergence check was performed on the mesh of the talus. With approximately two hundred thousand elements, the von Mises stress solution stability was reached. Because a small impingement was expected, a more refined mesh was used, consisting of 323,849, 322,986, 326,414 and 321,142 elements in the neck-like, pointy, round blunt and flat model respectively. Finally, we can obtain four intact FE models with different types of the posterior process of talus (Fig. 2C). All the FE models were constructed in the Cartesian coordinate system based on their anatomical positions (the y-axis is the sagittal direction of the model, while the z-axis is the axial direction).

### Model validation

A vertical downward load of 600 N along the z-axis was applied along the upper section of the tibia and fibula in ABAQUS 2020, which is in line with the in vitro experimental conditions by Anderson [13] and the FE study by Fan [14]. The peak stress and maximum contact area of tibiotalar articular cartilage were measured and compared to the in vitro experimental data [13]. The validation results were adequately close to the in vitro experimental data, which indicates that our model is real and reliable, and can be used for subsequent analysis.

### Ankle plantar flexion standing simulation

**Fig. 3:**
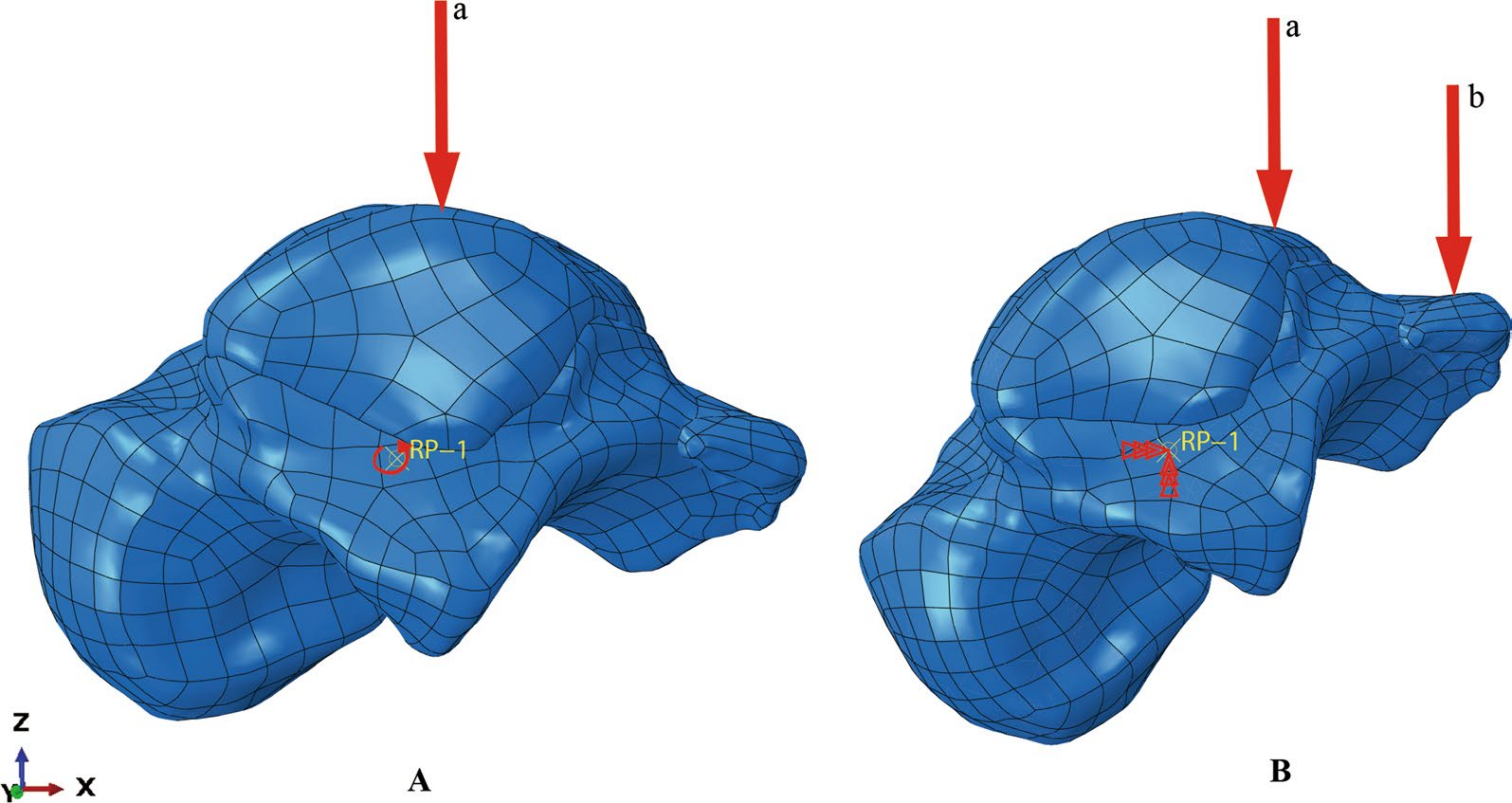
Boundary conditions. **A** Neutral position of the ankle joint (Initial position): An radius around the y-axis at the reference point-1 (RP-1) of the talus was applied under the compression force from the tibia and fibula (the red arrow marked “a”). **B** Hyperplantar flexion of the ankle joint: Impingement of the posterior process of the talus against the posterior malleolus (the red arrow marked “b”)

The mechanism of the fractures of PPOT can be a hyperplantar flexion of the ankle, with the posterior margin of tibia creating an impact on the PPOT [15]. Thus, this study simulated the single-leg standing in the ankle plantar flexion position. We assumed that a 70 kg subject is standing with only one leg on the ground, which means a total compression force should be 686 N. It was considered that a 642 N load is transmitted through the tibia, and 44 N is applied to the fibula according to the literature [16]. Figure 3 describes the specific boundary condition. To evaluate which type of the PPOT is most likely to fracture, the stress and strain of the talus were measured during the simulation in the four different models, respectively.

## Results

### The incidence and length of the PPOT

**Fig. 4:**
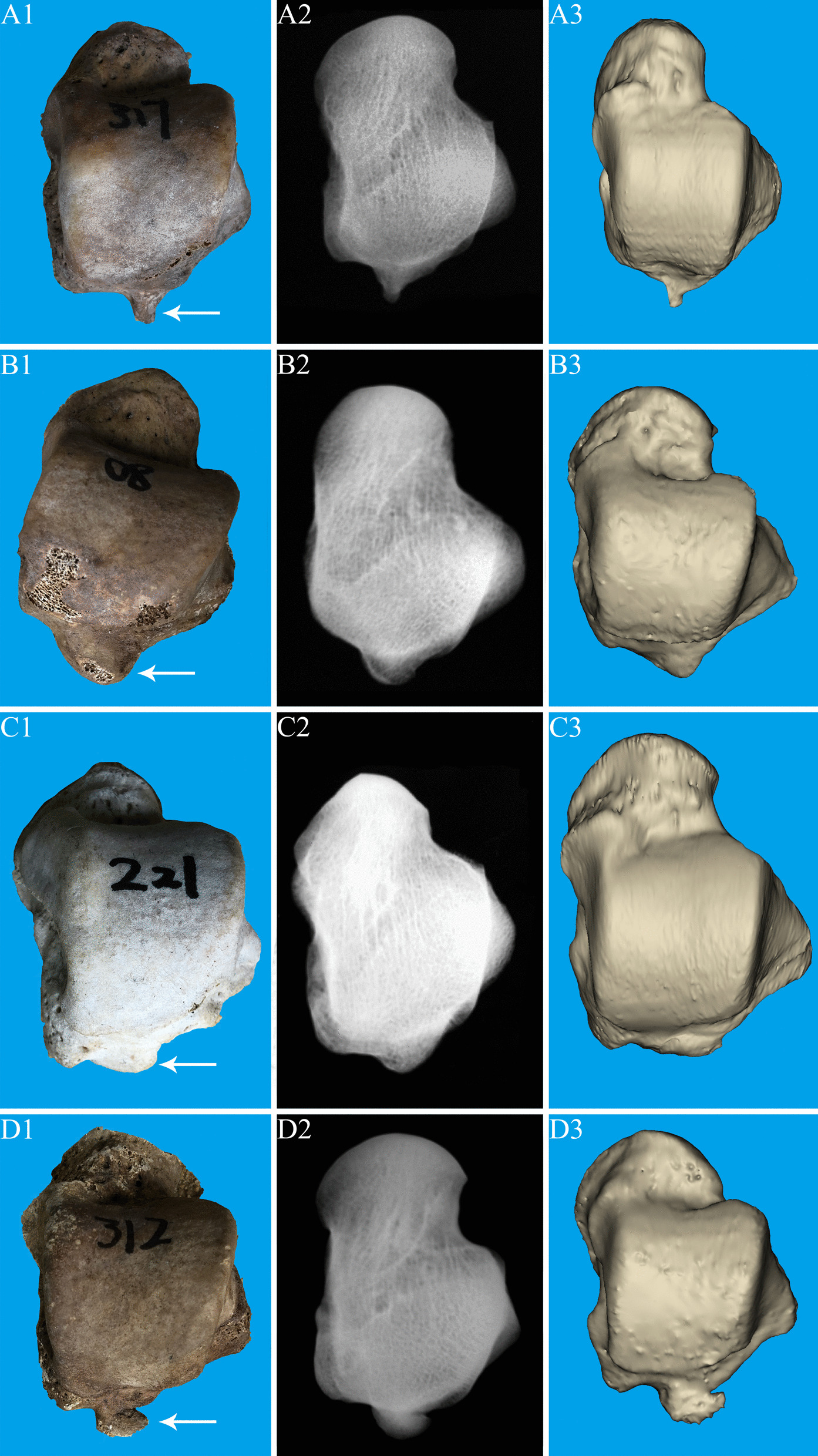
The types of the posterior process of the talus (anatomy, X-ray and three-dimensional reconstructed image). **A1–A3** Type 1: pointy type; **B1–B3** Type 2: round blunt type; **C1–C3** Type 3: flat type; **D1–D3**. Type 4: neck-like type

The overall incidence of the PPOT was 97.47% (462/474); the proportions of the types of the PPOT (Fig. 4) in the order of decreasing prevalence were as follows: the round blunt 39.31% (182/474), flat 37.15% (172/474), pointy 12.96% (60/474), and neck-like 10.37% (48/474) (Table2).

**Table 2:** Classification and incidence of the posterior process of the talus (n = 474)

Types	Neck-like	Flat	Pointy	Round blunt	None
Number of cases	48	172	60	182	12
Proportions	10.13%	36.29%	12.66%	38.39%	2.53%
The mean length of the medial tubercle (mm)	3.93 ± 1.12	3.43 ± 1.04	3.76 ± 1.19	3.97 ± 1.18	–
The mean length of the lateral tubercle (mm)	9.12 ± 1.65	6.54 ± 1.38	7.06 ± 1.52	7.82 ± 1.44	–
Number of bone cracks	11/48	2/172	3/60	7/182	–
Incidence of bone cracks	22.91%	1.16%	5%	3.84%	–

The mean length of the medial tubercle was 3.74 ± 1.22 mm (2.53–13.62 mm), and of the lateral tubercle was 7.37 ± 1.66 mm (2.53–13.62 mm). The mean lengths of the lateral tubercle of the types of the PPOT in the order of decreasing length were as follows: the neck-like (9.12 ± 1.65 mm), round blunt (7.82 ± 1.44 mm), pointy (7.06 ± 1.52 mm), and flat (6.54 ± 1.38 mm). The length of the tubercle on the medial side of the PPOT showed little difference among the four types (Table2).

### The incidence of bone cracks of the PPOT

**Fig. 5:**
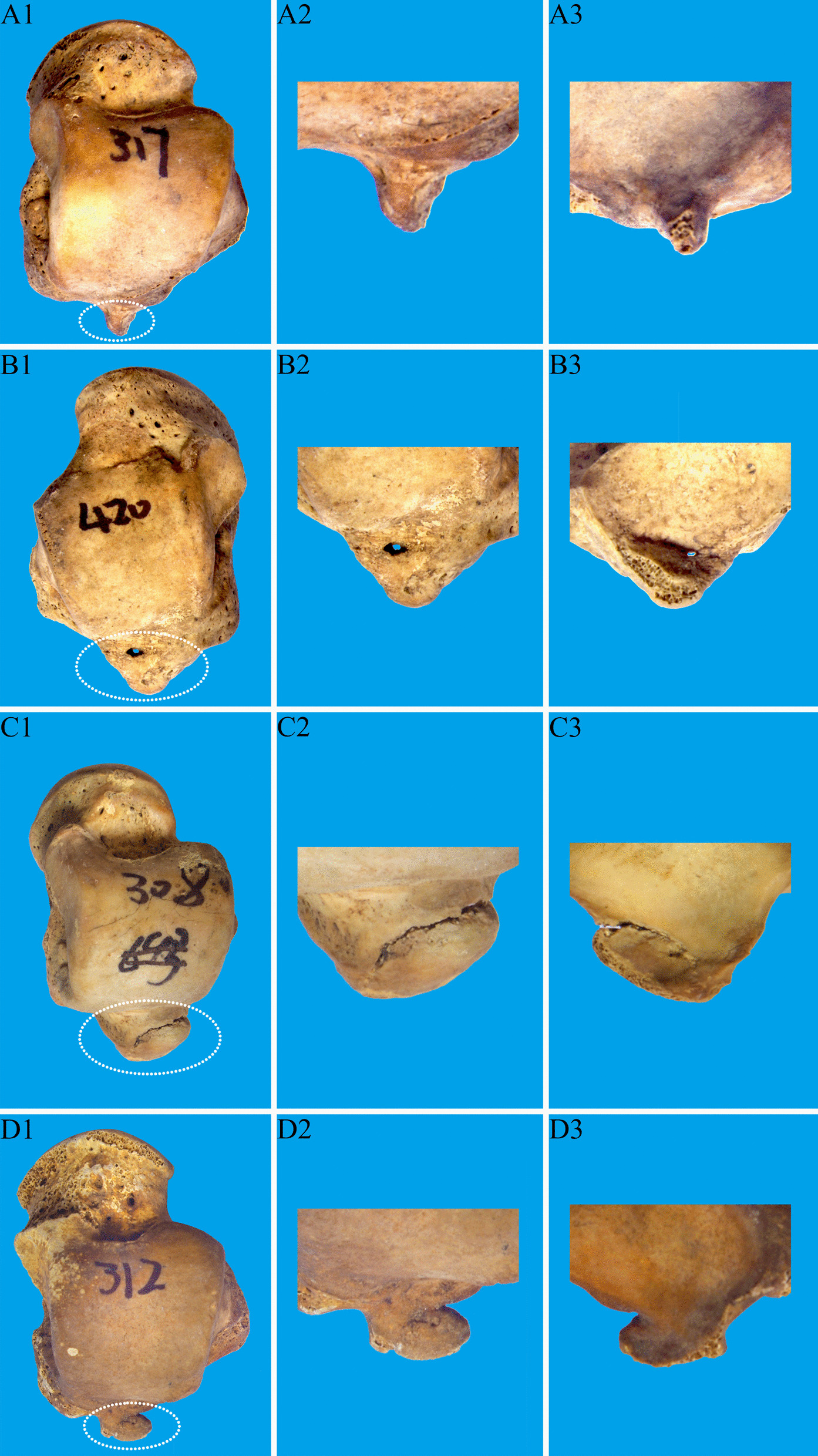
Fracture of posterior process of talus (overall, front and reverse partial anatomical view). **A1–A3**. Type 1: pointy type; **B1–B3**. Type 2: round blunt type; **C1-C3**. Type 3: flat type; **D1–D3**. Type 4: neck-like type

The overall incidence of bone cracks of the PPOT was 4.98% (23/462). The bone cracks of the types of the PPOT (Fig. 5) in the order of decreasing prevalence were as follows: the neck-like 22.91% (11/48), pointy 5% (3/60), round blunt 3.84% (7/182), and flat 1.16% (2/172), the neck-like type was observed the most bone cracks among these talus specimens (Table 2). And the bone cracks we found in the PPOT were all lateral tubercle.

### The stress and strain distribution of finite element (FE) models

**Fig. 6:**
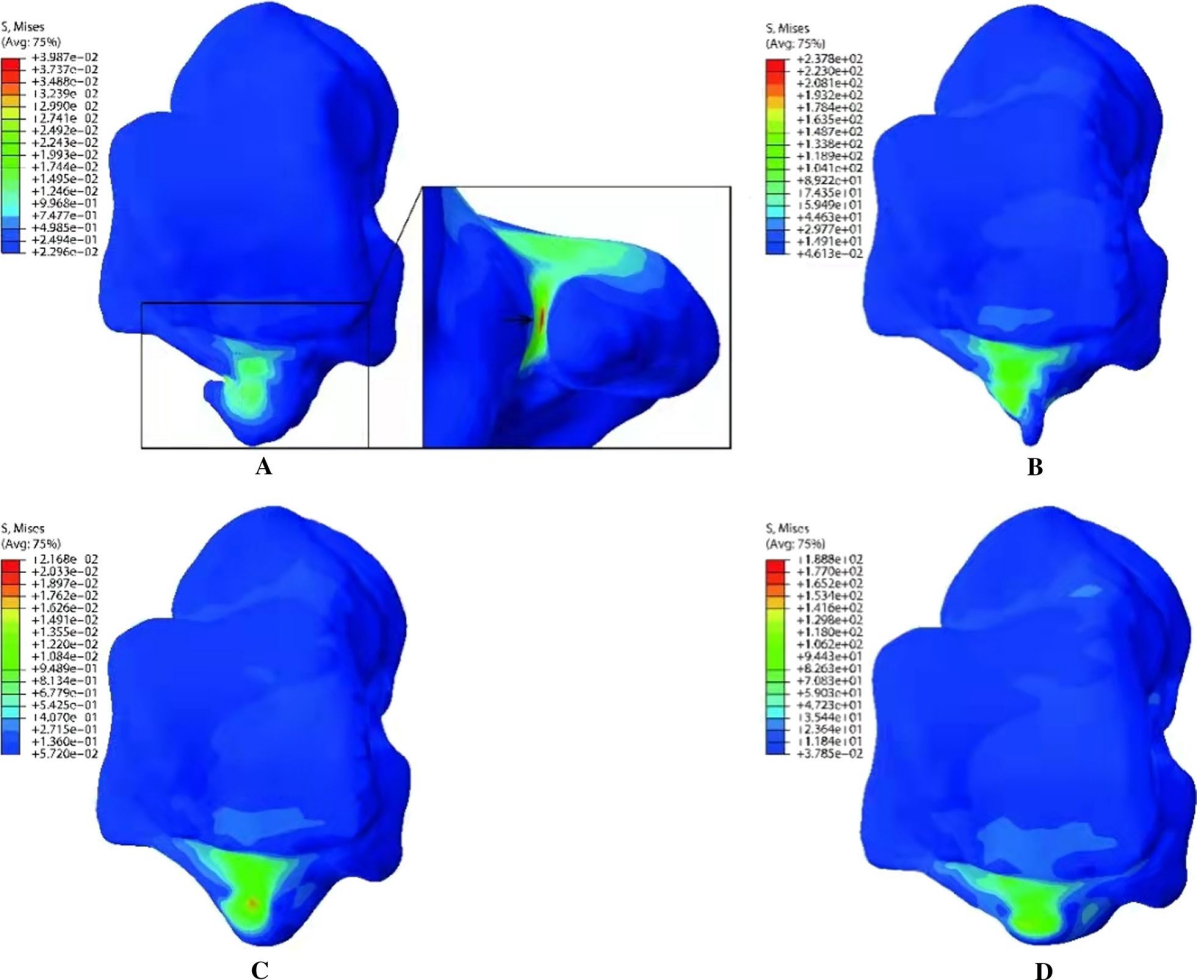
Stress distribution on the posterior process of talus. **A** Stress distribution on the neck-like posterior process. A long and narrow stress concentration area can be seen on the medial surface of the posterior process (black arrow); **B** Stress distribution on the pointy posterior process; **C** Stress distribution on the round blunt posterior process. **D** Stress distribution on the flat posterior process

The von Mises stress of the four models is mainly located on the lateral tubercle of the PPOT, because this study simulated a hyperplantar flexion of the ankle, which will make the posterior margin of tibia impact the lateral tubercle of the PPOT. We noticed the differences in the stress and strain distribution in the four different types of the PPOT. Compared with the value on the pointy, round blunt and flat posterior process of talus, the maximum von Mises stress increased by 67.66%, 83.90% and 111.18% on the neck-like posterior process of talus respectively. (Fig. 6) The von Mises stress was mainly located on the junction of the PPOT and talus body, which is also the location of fracture line observed on most of the talus specimens. In the neck like model, the von Mises stress was concentrated in a long and narrow area, which was located on the medial surface of the posterior process (black arrow in Fig. 6A) and was absent in the other three types of PPOT. To perform the convergence check, the mesh is refined in the region where the critical points are located. The strain distribution on the talus followed the stress distribution.

## Discussion

The fracture of the PPOT is a rare fracture in which the mechanical factors for fracture are not fully determined. Review of the literature demonstrates that fractures of the PPOT rarely involve the entire process, and isolated or lateral tubercle fractures are more common [17, 18]. This is consistent with our results that the fractures of the PPOT are mostly single fractures, and the fractures of the lateral tubercle are the majority. Fractures of the PPOT can be caused by indirect force ( such as sports injury or slipping off of steps) or direct force (will result from high velocity injuries such as vehicle accidents or high falls or high-speed sports) [19, 20]. According to the mechanism of injury and the fractures may be classified into three types: avulsion type, split type and comminuted type [20]. Indirect trauma can lead to avulsion or split fractures (mostly non-displaced), direct trauma may result in split fractures (often with displacement) or even comminuted fractures [3, 21, 22]. Specifically, the mechanisms of fracture are different for each type of fracture by indirect force. The lateral tubercle is considered to be caused by two mechanisms. Forced plantar flexion of the foot causes direct impingement between the posterior tibial plafond and the lateral tubercle; excessive dorsiflexion of the ankle can result in excessive tension of the posterior talofibular ligament, leading to an avulsion fracture of the lateral tubercle [21, 23]. There are also two main causes of medial tubercle fracture. Excessive plantarflexion varus of the ankle joint, the impact of the medial tubercle on the calcaneus, resulting in talus fracture; excessive dorsiflexion valgus of the ankle joint, excessive pull of the tibial posterior ligament of the deltoid ligament on the medial tubercle, resulting in avulsion fracture [8, 24].

According to our statistics, the proportion of cracks under the neck-like type lateral tubercles in the PPOT is much higher than that of the other three types, which may indicate that there is a greater possibility of fracture self-healing or inflammatory hyperplasia. In addition, the length of the lateral tubercles of the other three types is similar and shorter than that of this type, and the proportion of cracks is also lower. Furthermore, based on mechanical knowledge, the long and narrow stress concentration area shown in Fig. 4 is the vulnerable point of this model. We speculate that the reason for this phenomenon is associated with the morphology of the neck-like posterior process. The long and narrow stress concentration area is an important factor contributing to the neck-like posterior process fracture. Perhaps we can hypothesize that, with the same bone mass, the neck-like cervical segment may be subjected to more concentrated stress, leading to more fractures. This may be due to its shape (the lateral tubercle has a large head and a small middle) and the longer length of the lateral tubercle.

Most clinical ankle pain and injuries are straightforward ligamentous injuries. However, the clinical presentation of subtle fractures is similar to that of ankle sprains, and these fractures are frequently overlooked on initial examination [25]. The os trigonum is an accessory bone, frequently found in association with the posterolateral tubercle and believed to arise from a failure of fusion of a secondary center of ossification [23]. In our study, there were 12 samples with no obvious posterior protrusion and smooth posterior edge, which inferred the presence of os trigonum, and the incidence is about 2.53%. In the diagnosis of posterior process fracture of talus, it is easy to misdiagnose the fracture as irregular os trigonum [26]. Further examination with radiological findings may be required to distinguish between these fractures. Standard lateral radiograph of the foot usually best visualizes the lateral tubercle and the os trigonum [25]. Ebraheim et al. improved the X-ray examination method, using two strabismus images of 45° and 70° external rotation of the ankle joint to fully expose the medial tubercle, appropriately reduce the examination errors [27]. However, the radiological features of minimal cortical breach and subtle lucency are not always easily identified [5, 28]. CT or MRI is usually used to evaluate the anatomical details of foot and ankle bone structures and detect fractures, displacement, comminution and related injuries and osteochondral injuries [29].

There is no consensus on the best treatment for PPOT fractures. However, the treatment and management is aimed at restoring the anatomy of the talus and articular surface to maintain movement and stability of the ankle joint [5]. Undisplaced fractures can be treated conservatively, the fractures with an extraarticular or undisplaced small avulsion fragments are better treated for 6 to 8 weeks with cast fixation and partial weight bearing [30]. If symptoms persist for more than 3–4 months after injury, surgical intervention is needed [23, 31]. Displaced fractures should be treated surgically with arthroscopic exploration, surgical open reduction, internal fixation, or removal of fracture fragments, depending on the size of the fracture, to minimize long-term pain [4, 5]. Detailed treatment strategies for the PPOT fracture in previous literature are summarized in the first half of Table 3 [32, 33], but these strategies have little reference to the anatomical morphology classification of the PPOT.

**Table 3:** Management strategy for fractures of the posterior process of the talus

Fracture characteristics	Management	Fracture types		Management
< 0.5 cm, extra-articular, or avulsion fracture	Conservative	Pointy	Undisplaced	Conservative
< 0.5 cm, displaced, intra-articular	Arthroscopic excision		Displaced	Excision
0.5–1 cm, displaced	Arthroscopic assessment of chondral lesion	Flat	Follow the management strategy for fracture characteristics described above	
> 1 cm single fragment	Arthroscopic assisted screw fixation	Neck-like	Displaced neck is too thin to fixed	Excision
> 1 cm or multiple segmental fractures, or comminuted fractures, or fractures with dislocation	Emergency reduction, open reduction and internal fixation		Others	Follow the management strategy for fracture characteristics described above
		Round blunt	Follow the management strategy for fracture characteristics described above	

According to our experimental observation, it was found that among the types of bone cracks in the posterior process of talus, the most common type was neck-like type, followed by pointy type. We believe that different anatomical shapes of the PPOT may lead to different fracture types, and different fracture treatment schemes may be developed according to different fracture types (Table 3). For neck-like PPOT fractures, surgical excision should be performed regardless of the size of the displaced bone block if the neck fracture is displaced and surgical internal fixation is not possible. Even a small amount of displaced debris may damage the surface of the joint, causing obvious symptoms for pointy PPOT fractures, and these types of fractures can be treated with ankle arthroscopy and surgical excision. However, these views need to be supported by more basic research and clinical case reports in the future.

### Study limitations

Traditional anatomical morphology observation has limitations to a certain extent, and more clinical cases are needed for further exploration. In the finite element model, we assumed that the bones were homogeneous, continuous and isotropic, which might not be exactly match the reality. We used the von Mises stress failure criterion for bones, although it was not proven as a reliable measure for bones. But this can be a direction for our future research. We validated our model through comparison with a published in vitro experiment. Due to limited experimental conditions, we chose the peak stress and the maximum contact area as parameters for the model validation. Nevertheless, it is unlikely to alter our findings, in that the results for the traditional anatomical morphology observation and the finite element analysis support each other, which might have certain reference value.

## Conclusions

In our study, the PPOT was classified according to the anatomical morphology observation. Through the measurement and statistics of the classification and finite element models simulation of the PPOT, it is speculated that different types may be related to the probability of fracture of the PPOT. In the clinical treatment of PPOT fractures, it may be better to consider different treatment strategies for different types of fractures.

## Data Availability

The datasets used and analyzed during the current study are available from the corresponding author on reasonable request.

## Abbreviations

PPOT: Posterior process of the talus
CT: Computed tomography
FE: Finite element
NURBS: Nonuniform rational B-splines
3D: Three-dimensional
